# Why Septic Patients Remain Sick After Hospital Discharge?

**DOI:** 10.3389/fimmu.2020.605666

**Published:** 2021-02-15

**Authors:** Raquel Bragante Gritte, Talita Souza-Siqueira, Rui Curi, Marcel Cerqueira Cesar Machado, Francisco Garcia Soriano

**Affiliations:** ^1^ Interdisciplinary Post-Graduate Program in Health Sciences, Cruzeiro do Sul University, Sao Paulo, Brazil; ^2^ Immunobiological Production Section, Bioindustrial Center, Butantan Institute, São Paulo, Brazil; ^3^ Department of Emergency Medicine, University of São Paulo, São Paulo, Brazil; ^4^ University Hospital, University of São Paulo, São Paulo, Brazil; ^5^ Internal Medicine Department, School of Medicine, University of São Paulo, São Paulo, Brazil

**Keywords:** inflammation, septicemia, post sepsis syndrome, metabolic reprogramming, leukocytes

## Abstract

Sepsis is well known to cause a high patient death rate (up to 50%) during the intensive care unit (ICU) stay. In addition, sepsis survival patients also exhibit a very high death rate after hospital discharge compared to patients with any other disease. The addressed question is then: why septic patients remain ill after hospital discharge? The cellular and molecular mechanisms involved in the high rate of septic patient deaths are still unknown. We described herein the studies that investigated the percentage of septic patients that died after hospital discharge ranging from 90 days up to 5 years. We also reported the symptoms of septic patients after hospital discharge and the development of the recently called post-sepsis syndrome (PSS). The most common symptoms of the PSS are cognitive disabilities, physical functioning decline, difficulties in performing routine daily activities, and poor life quality. The PSS also associates with quite often reinfection and re-hospitalization. This condition is the cause of the high rate of death mentioned above. We reported the proportion of patients dying after hospital discharge up to 5 years of followed up and the PSS symptoms associated. The authors also discuss the possible cellular and metabolic reprogramming mechanisms related with the low survival of septic patients and the occurrence of PSS.

## Introduction: Sepsis Definition and Costs

Sepsis is a systemic organ dysfunction associated with unregulated host response to infection (1, 2). Septic shock is a subset of sepsis in which underlying circulatory and cellular/metabolic abnormalities are profound enough to substantially increase mortality. Patients with septic shock can be identified with persisting hypotension requiring vasopressors to maintain MAP ≥65 mm Hg and having a serum lactate level >2 mmol/L (18 mg/dl) despite adequate volume resuscitation.

The unregulated inflammatory response and consequent loss of generalized organ function in septic shock can lead the patient to death. Hospital mortality of patients with septic shock is more than 40% (2). Sepsis is widely recognized as a highly life-threatening condition associated with a high rate of patient deaths during intensive care unit (ICU) stay in the whole world (3).

Several pathogens can cause sepsis; bacterial or fungal infections represent the majority of cases. Despite this fact, negative blood culture for microorganisms has a prevalence of up to 42% of septic patients (4). There are few reports on viral infections causing sepsis. The incidence of viral sepsis is not precisely known, and there is insufficient information to make an accurate estimate; diagnosing viral sepsis is very rare. It has been reported that almost all viruses can cause sepsis when the patient has an impaired immune function (4). The SPREAD study (Sepsis Prevalence Assessment Database) reported about two hundred ninety cases of sepsis per 100 thousand populations in Brazil, with a mortality of up to 55%. Septic patients occupy about one-third of intensive care units (5, 6).

In 2017, global estimates sepsis incidence and mortality indicated 48,9 million new cases worldwide. The number of deaths reached up to 11 million, representing 19.7% of the patients (7). The expenses ranged from US$ 24,638 to US$38,298 per patient in the USA (8), from 23,000 to 29,000 € in Europe (9), and around US$ 9,600 in Brazil (10).

There is substantial knowledge of long-term sepsis symptoms and causes of death (11). However, the causes of sequelae in patients after sepsis remain unknown yet. Patients recovered from sepsis exhibit impaired immune function and a chronic inflammation state, similarly to elderly and immunosuppressed patients with inadequate response to infections (11)

Despite the information, the evolution of septic patients’ survival after hospital discharge has drawn the attention of researchers worldwide. After curing sepsis and hospital discharge, septic patients’ mortality is much higher than that of patients from any other disease.

We described the studies that investigated the percentage of septic patients that died after hospital discharge ranging from 90 days up to 5 years. We also reported the symptoms of septic patients after hospital discharge and the development of the post-sepsis syndrome (PSS) characterized by several clinical manifestations.

## Methods

We conducted a literature search on MEDLINE in PubMed, using relevant search terms and synonyms for sepsis, survivors, and long-term mortality, and excluded non-English language articles. We searched for retrieved studies to find other relevant data: clinical trials, large longitudinal, and observational studies. We summarized recent insights into the outcome of septic patients after hospital discharge.

## The Rates of Death of Septic Patients After Hospital Discharge

**Table 1** shows the reports on this matter by several research groups. The authors described a substantial amount of patients who died as early as 90 days of the hospital discharge and during a follow up of 5 years. Next, we describe the literature data grouped by time of follow up: 1, 2, and 5 years.

**Table 1:** Percentage of death of septic patients after hospital discharge.

Authors	Number of patients	Country	Up to 90 days	Up to 1 year	Up to 2 years	Up to 5 years	Age (years)
Quartin, Schein (12)	1.505	USA	NI	66%	NI	100%	61,7 ± 12.6
Jagodic and Podbregar (13)	164	Slovenia	NI	30%	70%	NI	64 ± 13
Karlsson, Ruokonen (14)	470	Finland	NI	NI	44,9%	NI	59,6 ± 15,1
Iwashyna, Ely (15)	1.194	USA	41.3%	NI	NI	~82%	76,9 ± 8,7
Cuthbertson, Elders (16)	439	Scotland	NI	NI	NI	61%	45 a 67
Prescott, Langa (17)	1.083	USA	27.5%	44.2%	NI	NI	78,6 ± 8,6
Wang, Szychowski (18)	30.239	USA	NI	23%	~29%	~44%	≥45
Davis, He (19)	1.092	Australia	NI	12.5%	22.4%	NI	46,9 ± 17,3
Prescott, Osterholzer (20)	–	USA	35.3%	48.5%	56.5%	NI	≥65
Shankar-Hari, Harrison (21)	94.748	England	NI	15%	8.3%	21.1%	61,3 ± 17,0
Yende, Kellum (22)	483	USA	~9%	~30%	NI	NI	60,5 ± 15,2
Courtright, Jordan (23)	87.581	USA	NI	~28%	NI	NI	≥75

NI, Not informed.

Prescott, Langa (17), Yende, Kellum (22), and Courtright, Jordan (23) conducted cohort studies in septic patients. The authors had the main objective of assessing the mortality rate after one year of hospital discharge. The studies included 1,083, 483, and 87,581 patients, respectively. The three studies showed similar results, with a death rate between 28 to 44%. Yende, Kellum (22) demonstrated that in addition to the high mortality rate, survivors showed a persistent increase in the blood levels of inflammation and immunosuppression biomarkers associating these observations with worse long-term outcomes. Courtright, Jordan (23) also pointed out that 68.2% the patients who died were re-hospitalized in the last 30 days of life.

Jagodic and Podbregar (13), Karlsson, Ruokonen (14), Davis, He (19), and Prescott, Osterholzer (20) carried out observational studies intending to determine short and long-term survival, besides, to evaluate the quality of life of septic patients after the hospitalization period. The researchers studied 164, 470, and 1,092 patients, respectively. They concluded that septic patients have a high mortality rate after two years of hospital discharge compared to patients recovered from other diseases; the percentage of death ranged from 22% to 70%. They also reported a marked reduction in the survivors’ quality of life.

Quartin, Schein (12), Iwashyna, Ely (15), Cuthbertson, Elders (16) and Wang, Szychowski (18) performed cohort studies to determine long-term mortality in septic patients. The researchers investigated 1.505, 1.194, 439, and 30.239 patients, respectively. The authors reported that the mortality rate varied between 44% and 100% of the total survivor’s septic patients after five years of hospital discharge. It is remarkable that in many cases there were no septic patients alive in the short period of five years after hospitalization. There is a possibility that the sepsis promotes an early aging state in the post-sepsis patients. This assumption is supported by the fact that aging markers such as telomeres shortage are increased in patients with sepsis (24).

## Post-Sepsis Syndrome (PSS)

There is an association between preexisting comorbid conditions and some of the long-term outcomes following sepsis demonstrated by robust statistical methods. However, epidemiological causality and biological mechanisms are not fully established yet.

Charlson’s Comorbidity Index allows determining the impact of comorbidities in the outcome of patients with severe infections or sepsis (25, 26). The weighted Charlson’s Comorbidity Index assesses chronic diseases’ presence to predict 10-year survival in patients with multiple comorbidities (27). There is also a high prevalence of comorbidities in post-septic patients - most studies listed in **Table 1** report comorbidities (12, 13, 17, 18, 21, 23).

The post-sepsis-syndrome cognitive decline relationship is complex and bidirectional. Pre-illness cognitive decline is a risk factor for pneumonia and sepsis. On the other hand, sepsis is an independent risk factor for cognitive function decline (OR 95% CI 3.3 (1.5–7.3) (28, 29). Other authors found that sepsis *per se* confers additional risk to late mortality predicted by status before sepsis itself (20). In fact, sepsis more often occurs in patients with preexisting chronicle conditions (14, 30), for instance, frail and elderly (10).

In a previous study, we postulated that in elderly with sepsis, in addition to comorbidities, a combination of factors including intestinal barrier dysfunction and dysbiosis, DNA damage, mitochondrial dysfunction, telomere shortening, and epigenetic mechanisms have synergistic effects contributing to a worse outcome (31).

The diverse clinical manifestations in the post-septic patients have been named altogether as post-sepsis syndrome-PSS. The PSS syndrome includes various clinical manifestations such as changes in metabolism; for instance, a decrease in total body protein content, high retention volume of fluid, prolonged time for returning to normal hydration, and an increase in total energy expenditure (32–34). Several studies showed that post-sepsis syndrome also includes immune function impairments such as a persistent inflammatory state characterized by an increase in High mobility group box 1 (HMGB1) levels in plasma after sepsis (35–37). HMGB1 is a protein of the nucleus and binds to DNA acting as a co-factor for the transcription of certain genes. HMGB1 released into the extracellular fluid exhibits pro-inflammatory properties by working as a damage-associated molecular pattern molecule (DAMP). HMGB1 triggers innate immune responses through activation of various cell surface receptors; the receptor for advanced glycation end-products (RAGE) and toll-like receptors(TLRs); TLR2, TLR4, or TLR9 (38).

**Figure 1:**
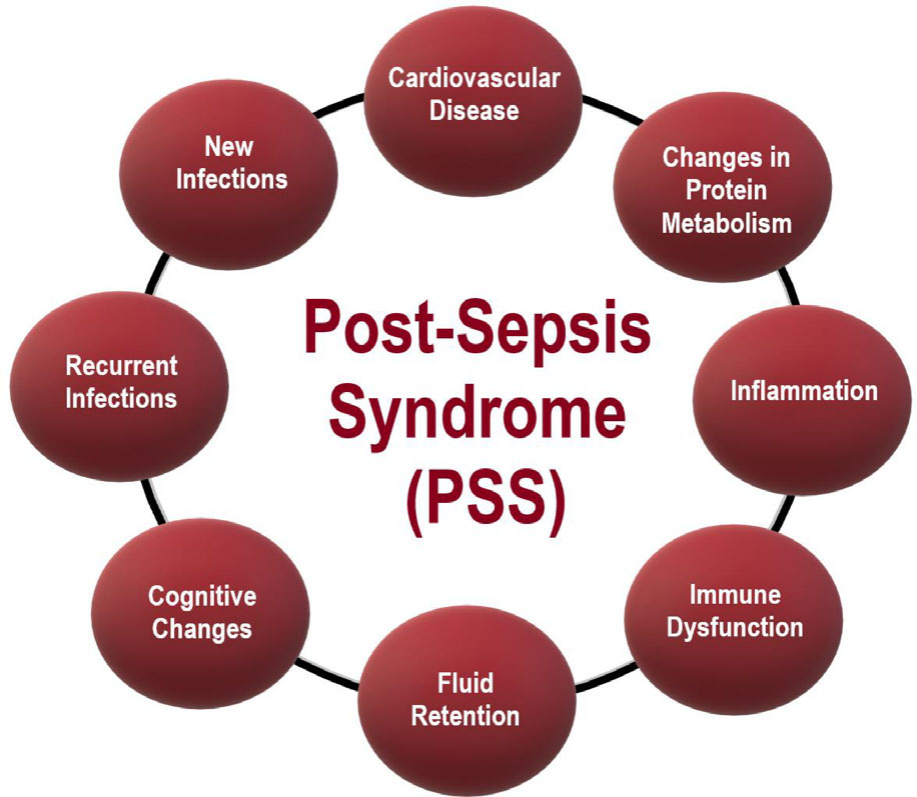
Post-Sepsis Syndrome – The most important symptoms.

Monneret and Venet (37) described that neutrophils have impaired chemotaxis and oxidative burst activities. The authors also reported an increased number of circulating immature neutrophils. There is also evidence for impaired lymphocyte and natural killer cell functions. Sepsis survivors had a high level of re-hospitalizations, caused by pneumonia (18, 39–43) (including sepsis again). PSS also is characterized by cognitive sequelae, such as dementia (44–50) and changes in the cardiovascular system and cardiovascular diseases (51–55). These changes lead to a decrease in the quality of life of the pots-sepsis patients, with psychological alterations (e.g., anxiety, memory loss), and mental problems (incapability of doing easy calculations for instance) (56). **Table 2** reports the symptoms post-sepsis patients more often exhibit. A summary of the PSS symptoms herein reported is in **Figure 1**.

**Table 2:** Sequels in septic patients after hospital discharge.

	Sequels	Authors
**Causes of Re-hospitalization**	Occurrence of infection and pneumonia	Wang, Derhovanessian (39)
	Infection (unresolved/recurrent or new infections)	Sun, Netzer (40)
	Renal dysfunction and urinary tract infection	Zilberberg, Shorr (41)
	Recurrent infections	Wang, Szychowski (18), Yende and Angus (42), Linder, Guh (43)
**Cognitive** **Changes**	Dementia	Shah, Pike (44)
	Depression	Davydow, Hough (45)
	Delirium	Tsuruta and Oda (46)
	Moderate or severe cognitive depression	Angus (47)
	Moderate to severe cognitive impairment; Functional disability	Iwashyna, Cooke (48)
	Reduction on physical, sensory, emotional and cognitive functioning	Lazosky, Young (49)
	Reduction on physical function	Poulsen, Møller (50)
**Cardiovascular Diseases**	Cardiovascular disease	Yende, Linde-Zwirble (51)
	Myorcadial infarction	Dalager-Pedersen, Søgaard (52), Mejer, Gotland (53), Ou, Chu (54)
	Brain Stroke	Wu, Tsou (55)
**Changes in Metabolism**	Decrease in total body protein, High retention volume of fluid, Prolonged time for returning to normal hydration, Increase in total energy expenditure.	Brun-Buisson, Doyon (32)
	Uremia and hyperglycemia	De Deyn, Vanholder (33)
	Hypoxia, hyperglycemia, increased uremia, and plasma ammonia levels.	Sonneville, Verdonk (34)
**Inflammation and Immune** **Dysfunction**	Persistent inflammatory state, high HMGB1 in plasma after sepsis.	Chavan, Huerta (35)
	DNA methylation of monocytes with tolerable phenotype	Lorente-Sorolla, Garcia-Gomez (36)
	Neutrophils: impairment of chemotaxis and oxidative burst, and increased number of circulating immature cells.	Monneret and Venet (37)
	Macrophages: impairment of antigen presentation and in the production of pro-inflammatory cytokines	Monneret and Venet (37)
	Lymphocytes (CD4/CD8): decreased number, reduced IFN-γ production, phenotype changes, and decreased proliferation of regulatory T	Monneret and Venet (37)
	Natural Killer cells: impaired production of IFN-γ and decreased cytotoxicity activity.	Monneret and Venet (37)
**Quality of Life**	Lower physical activity functioning, decrease on vitality, role-emotional, mental health, and mental component scores.	Zhang, Mao (30)
	SF-36 lower	Cuthbertson, Elders (16)
	SF-36 lower, physical component score (CS) lower until 6 months after sepsis.	Nesseler, Defontaine (57)
	Patients could not live independently, problems in mobility, usual activities and self-care domains.	Yende, Austin (58)

SF-36, Short-Form Health Survey; HMGB1:High Mobility Group Box 1 protein.

Prescott, Langa (17) reported that of 2,617 septic patients survivors, 40% were admitted to the hospital again after 90 days. Readmissions were mainly due to a recurrence of sepsis (11.9%), and also due to pneumonia and urinary tract or skin or soft tissue infections. The incidence of these infections was much higher in post-septic patients than patients who survived from other diseases.

In a study of 93,862 septic patients, one year after hospital discharge, survivors had a higher risk of developing cardiovascular events, such as ischemic and hemorrhagic stroke, myocardial infarction, and heart failure. The authors followed the patients for 5 years and reported risks for patients during the whole period of study. Five years of follow up reported 15.3% of patients with a mental decline, emotional, and behavioral conditions; 50.2% of those were re-hospitalized (54), 27.2% needed respiratory treatments, and approximately 46% had declined on cognitive function (23).

Several studies use the Short Form Health Survey (SF-36) to evaluate the patients’ quality of life (44, 59, 60) through questions covering functional capacity, physical aspects, pain, general health, vitality, social issues, emotional elements, and mental health (46, 59). Sepsis or septic shock survival patients exhibit a poor and sometimes very poor quality of life (61). Hofhuis, Spronk (28) reported that even three months after hospital discharge, physical functioning recovery is incomplete (62). This condition persists until two years or more (59).

Septic patients do develop several limitations after leaving the hospital. It has been reported difficulties in performing straightforward daily activities, managing money, taking bathe, or using the toilet. These physical activity limitations are associated with myopathy, neuropathy, or cardio-respiratory problems, independently or together (15). Patients also have lost sensory, digestive, and kidney dysfunctions, skeletal muscle pain, and chest discomfort. Huang, Daniels (63) reported that of 1,731 patients, only 5.5% said they felt very well after sepsis. Most of them reported feeling worse or different from the conditions before the sepsis. The decline in the ability to read, spell, and libido occur concomitantly with anxiety, depression, and sleep disorders.

## Discussion: Inferences and Investigation Directions

The massive infection and the accompanying intense immune response with a cytokine outpouring during sepsis may promote irreversible cell metabolic reprogramming. The cell reprogramming is unlikely to occur in leukocytes or bone marrow only. These might happen in several tissues and cells that prompt to systemic organ dysfunctions. The cell metabolic reprogramming persists even after hospital discharge and might be associated with the PSS symptoms. In other words, a septic patient no more returns to the health state before the infection and hospitalization.

The molecular mechanisms associated with cell metabolic reprogramming in sepsis deserve investigation. Bacteria can transfer genetic material to host cell DNA (64) as the eukaryotic cells develop tools to protect themselves against the microorganism invasion (65). The latter may induce cell biology and metabolic reprogramming that remains even after the infection elimination. Particularly, miRNAs formed during sepsis can define a repertoire of gene expressions reprogramming different cells and tissues. This postulated molecular mechanism may explain the fact that PSS involves marked manifestations in widespread organs. Cell reprogramming might also involve epigenetic mechanisms such as DNA methylation and histones methylation or acetylation.

Leukocytes (macrophages, lymphocytes, and neutrophils) functions depend on the heterogeneity and the plasticity properties of these cells. Several research groups documented the plasticity of leukocytes in normal and disease conditions (66–68). All leukocytes have a recognized ability to sense the microenvironment and modify their cell biology and metabolism to accomplish the organism’s requirements for that specific moment accordingly (69). Leukocytes can reprogram the intracellular metabolism quickly and efficiently to respond to inflammatory or infectious stimuli generating a different phenotype (70). The metabolism fate of leukocytes is closely associated with the phenotypes of these cells. The metabolic reprogramming is part of the leukocyte phenotyping plasticity.

Macrophage functions play a crucial role in the homeostasis, including involvement in inflammatory and immune responses. There are two macrophages types; (M1) pro- and (M2) anti-inflammatory macrophages (66). The polarization of macrophages involves marked changes in the intracellular metabolic pathways (71). M1 macrophages exhibit a high glycolytic activity that generates the required ATP in the cytosol, whereas M2 macrophages use the Krebs cycle and the mitochondrial oxidative phosphorylation (OXPHOS) for energy production (72, 73).

Sepsis causes marked changes in macrophage functions and increases M1 cell proportion, particularly in the acute phase. Macrophage metabolism reprogramming during sepsis (74–78) may remain after hospital discharge. On the other hand, sepsis may generate a specific macrophage phenotype (a suggested Msepsis monocyte with sepsis memory still to be investigated) that remains active up to three years after hospital discharge. In a previous study, we identified monocyte changes from septic patients even three years after ICU discharge by assessing mRNA expression of inflammatory mediators and monocyte polarization, indicating macrophage reprogramming (data not published).

Cell metabolism reprogramming is also involved in the functions and even generation of the different lymphocyte subsets. Several stimuli and conditions change lymphocyte metabolism (79), including microenvironment nutrient availability (80). The T helper lymphocyte (Th) maturation involves particular function acquisitions that result in the generation of Th1, Th2, and Th17 subsets. These effector T cells, particularly Th17, can generate other phenotypes under specific stimuli and disease conditions (81). It is remarkable to discover that glycolysis inhibition using 2-deoxyglucose, a non-metabolizable compound that enters into the cells, converts the Th17 cells into Treg cells (82). Th17 lymphocytes can also generate non-classical Th1 cells with particular properties different from the classical ones. The mentioned Th17 plasticity may play an essential role in host protection and chronic inflammatory conditions (83). Metabolic reprogramming of the Th17 lymphocytes or even the generation of a particular lymphocyte subset in sepsis remains in question marks.

Another type of lymphocyte is Treg cells, responsible for maintaining immune self-tolerance and controlling autoimmune responses (84). Treg lymphocytes are essential regulators of the inflammatory response through secretion of cytokines that inhibit the action of effector T lymphocytes (CD4 + and CD8 +), culminating in the gradual decrease of the inflammatory response without harming the antigen response (84–86) reported that on the first day of septic condition, the percentage of Treg lymphocytes is higher in patients who survive than patients who die. The role of Treg lymphocytes in the PSS remains uninvestigated.

There are a large number of neutrophil phenotypes and functions in different tissues in health and diseases. Yang, Li (87) and Silvestre-Roig, Fridlender (88) reviewed the neutrophil phenotypes and tasks in various conditions such as inflammation and rheumatoid arthritis. Neutrophil kills microbes and exhibits high plasticity to accomplish its functions in situations of limited metabolite availability. We then speculate that a new subpopulation (to be described) of neutrophils (a suggested Nsepsis neutrophil with sepsis memory still to be investigated) develops and may remain even after hospital leaving during a sepsis or septic shock (24).

We presented herein directions for research to address the fact that septic patients remain sick after hospital discharge.

## Study Limitations

This study has some limitations. We reviewed only English language articles and articles directly related to the subject of this study. Articles not directly related to the subject of the present study may be missing. We did not establish the relationship between comorbidities and the late outcome after sepsis due to the lack of information.

## Data Availability Statement

The original contributions presented in the study are included in the article/supplementary material. Further inquiries can be directed to the corresponding author.

## Author Contributions

Conceived and designed the Review: RG, TS-S and RC. Contributed to the preparation of the tables: RG, TS-S, RC, MM, and FS. Wrote the paper: RG, TS-S, RC, MM, and FS. Final approval of the version to be submitted: TS-S, RC, MM, and FS. All authors contributed to the article and approved the submitted version.

## Funding

FAPESP, CNPq, CAPES, the Guggenheim Foundation, University Hospital of the University of Sao Paulo, University of São Paulo Medical School, Sirio Libanes Hospital of São Paulo, and Cruzeiro do Sul University financially support the research studies of our groups.

## Conflict of Interest

The authors declare that the research was conducted in the absence of any commercial or financial relationships that could be construed as a potential conflict of interest.
